# BET degrader inhibits tumor progression and stem-like cell growth via Wnt/β-catenin signaling repression in glioma cells

**DOI:** 10.1038/s41419-020-03117-1

**Published:** 2020-10-22

**Authors:** Tao Tian, Tongqi Guo, Wei Zhen, Jianjun Zou, Fuyong Li

**Affiliations:** 1 Department of Oncology, Shandong Zaozhuang Municipal Hospital, Zaozhuang City, Shandong Province China; 2 Department of Neurosurgery, The People’s Hospital of China Medical University (The People’s Hospital of Liaoning Province), Shenyang, Liaoning Province China

**Keywords:** CNS cancer, Pharmaceutics

## Abstract

Based on their histological appearance, gliomas are a very common primary tumor type of the brain and are classified into grades, Grade I to Grade IV, of the World Health Organization. Treatment failure is due to the cancer stem cells (CSC) phenotype maintenance and self-renewal. BET degraders such as ZBC260 represents a novel class of BET inhibitors that act by inducing BET proteins degradation. This study explores the mode of action and effects of ZBC260 in vivo and in vitro against glioma. By inhibiting cell proliferation and inducting cell cycle arrest, the fact that glioma cell lines show sensitivity to ZBC260. Notably, ZBC260 targeted glioma without side effects in vivo. In addition, the stem cell-like properties of glioma cells were inhibited upon ZBC260 treatment. When the mechanism was examined, our findings indicated that Wnt/β-catenin pathway repression is required for ZBC260-induced stem cell-like properties and tumor growth suppression. In conclusion, the growth of tumors and stem cell-like properties were inhibited by ZBC260 via Wnt/β-catenin repression, which suggests ZBC260 as a potential therapeutic agent for glioma.

## Introduction

Glioma is a malignant primary brain tumor owing to brain and spinal glial cell carcinogenesis^1,2^. According to The world health organization (WHO), pathologically gliomas are classified as Grades I and II are LGGs or low-grade gliomas. Grades III and IV are classified as HGGs, or high-grade glioma, with Grade IV also known as GBM or glioblastoma multiforme, or just glioblastoma^3,4^. Glioblastoma multiforme was prevalent in 16% of all primary brain tumors with as much as 54% share in all gliomas, marked by high mortality, high rate of recurrence, an extremely low recovery rate as well as high morbidity and recurrence rates^5,6^. Even with the palpable advancement over the decades, in adjuvant therapy and noticeable evolution of surgical techniques and technology advancements, prognosis, as well as the treatment of gliomas still pose extraordinary challenges^1,7^. The current standard therapy for glioma is surgical removal, with concomitant chemoradiotherapy and adjuvant chemotherapy with temozolomide (TMZ) performed after the surgery^8,9^.

As an epigenetic reader, the bromodomain and extra-terminal domain (BET) protein identify and bind to acetylated lysine residues^10,11^. The BRDT protein that is restricted to the testis and which recognizes histones 3 and 4 lysines and some transcription factors, as well as the universally expressed BRD2, BRD3, and BRD4 bromodomain proteins, together make up the BET protein family^12,13^. The BET proteins have an important part in cancer especially as part of the units that regulate the proliferation, metabolism, elongation, metastasis and the transcription of cancer stem cells^14^. The BRD4 has a crucial function in the regulation of a vital oncogene prevalent in many types of tumors and is also a significant part of the organization of super-enhancers making it the most widely studied member of the BET protein family^12,15^. In anti-cancer drugs, BET inhibitors are being used and the rationale behind developing and using these inhibitors are the preclinical studies of the role of BET proteins in cancer^16^. The BET inhibitors, bind the bromodomains in particular thereby not allowing the proteins of BET to bind with chromatin and as a result not allowing gene transcription^11,13^. While in the pre-clinical models, the BET inhibitors have displayed anti-cancer behavior broadly, the first-generation inhibitors have shown very average results clinically, most probably owing to the therapeutic index, which is quite narrow and does not include the ideal target engagement^17,18^.

Proteolysis targeting chimera (PROTAC) molecules are bound to the targeted proteins on the one side and the other side is recognized by E3 ligase that is Cullin dependent, and therefore, are bi-functional molecules^19,20^. The oncogenic proteins experience selective degradation by the PROTAC molecules, which utilize the ubiquitin proteasome system (UPS)^21,22^. Therefore, in the recent past, some researchers reported the synthesis of the pharmacological molecule BET-PROTAC^23^. BET proteins of the cancer cells are totally eliminated by the BET-PROTACs as has been shown in the pre-clinical trials^24,25^. However, in the case of glioma, the anti-cancer effect of BET-PROTACs has still not been studied. Basing on the BET inhibitor HJB-97, the newly synthesized ZBC260, is the all new BET-PROTAC^26–28^. In this report, we explain our investigation into the antitumor work of ZBC260, both in vitro as well as in vivo, with regard to glioma.

The signaling pathway Wnt/β-catenin is vital with respect to cell invasion, angiogenesis, migration, and proliferation and has a close relation to various tumorigenesis^29^. In the approved the Wnt/β-catenin pathway, upon activation of the Wnt signal, FZD (Frizzleds) gets bound by Wnt protein, according to Dvl (Disheveled) action, a huge volume of β-catenin gets stored as it phosphorylates, after which there is binding happening with lymphoid enhancer-binding factor/T-cell factor (LEF/TCF) transcription complex to conciliate the start of a series of target genes of Wnt including cyclin D1 and c-Myc, and therefore as a result regulate cell differentiation and proliferation^30,31^. Cell apoptosis and proliferation in glioma was caused by the abnormal reporting of Wnt/β-catenin signaling, and this led to the development of glioma^32^. An earlier study attributed self-renewal, resistance to radiation and stemness was related to the pathway of Wnt/β-catenin signaling^33^. Wnt5a, a significant member of the Wnt family, the has studies of it display that changes are related to the progression of the glioma^34,35^. Obviously, it is of great significance to explore the molecular mechanisms of β-catenin signaling pathways regulating glioma.

This study explores the mechanism of activity and effects of ZBC260 in vitro and in vivo against glioma. Such a study can ascertain the effectiveness of the molecule as an agent of therapy for glioma. Biomarkers of value for predicting prognosis in glioma patients can be uncovered by identifying targets of ZBC260.

## Materials and methods

### Culture of the Cell

Human glioma cell lines including U251, H4, U87, A172, U118, LN229, and SHG-44 were obtained from the American Type Culture Collection (ATCC, USA). Cells were cultured in Dulbecco’s Modified Eagle Medium (DMEM) (Gibco) supplemented with 10% fetal bovine serum (FBS: Gibco) along with streptomycin (100 mg/L) and penicillin (10,000 U/L) at 37 °C in a 5% CO_2_ humidified incubator. ZBC260, JQ1 and HJB-97 were obtained from MedKoo Biosciences, Inc. All of the above cell lines were authenticated by short tandem repeat profiling and were routinely tested for mycoplasma contamination. The dimethyl sulfoxide (DMSO) stock of ZBC260, JQ1 and HJB-97 were conducted that was proportionate to the concentrations with cell culture medium (DMSO final concentration < 0.1%).

### MTT assay

Ninety-six-well flat-bottom plates were seeded with 5000 cells (total) following exposure to various concentrations of ZBC260 (180 μL final volume) for 72 h. To each well, 5 mg MTT/mL (20 μL) in phosphate-buffered saline (PBS) was added at experimental time spans, and the plates were for 2 h at 37 °C. Crystals of formazan were solubilized with 150 μL DMSO following media removal. A microplate reader was used to determine absorbance at 490 nm.

### Analysis of cell cycle

Propidium iodide staining was performed on cells subjected to ZBC260 treatment at 37 °C in a 5% CO_2_ incubator for 24 h. This was followed by analysis on a Cytomics FC 500 instrument (Beckman Coulter), and within the cell cycle, the percentage of cells was arrived at using the cycle analysis software.

### Wound-healing assay

A 6-well plate was used to seed the cells, which were then incubated at 37 °C for the duration of time that was required to achieve ninety percent confluency. A sterile 20-μL tip was used to make a scratch and was then washed with PBS. After that, a DMEM (serum-free) was used to culture the cells for 24 h. The ImageJ software was used to measure the wound gaps and in three independent experiments the wounds were normalized to time zero.

### Assay for cell invasion

A 24-well invasion chamber system (BD Biosciences) was used to develop the cell invasion assay using a polycarbonate membrane of 6.5 mm diameter, 8 μm pore size, and pre-coated with Matrigel (Corning). Suspension of cell (1 × 10^5^ cells/mL) was readied in a serum-free medium, and 200 μL cell suspension was introduced into the upper chamber. After that the lower chambers were filled with 500 μL medium containing 10% FBS. After 24-h incubation at 37 °C, from the top of the membrane, and the non-invasive cells were scraped off. Ten percent formaldehyde was utilized to fix the filters and stained with crystal violet (0.1%) for 15 min. Enumeration of cells from the three light microscope fields chosen randomly, the cells were counted. Each assay was carried out in copies of three.

### Apoptosis assay

For flow cytometry, following 24 h of ZBC260 treatment, exposed cells were collected, followed by double washing with PBS. Staining with propidium iodide (PI) and Annexin V-FITC was carried out in the dark for 15 min. For assaying cell death, FACScan from Becton Dickinson (Franklin Lakes, NJ, USA) was used and data was analyzed by CellQuest software. The total number of cells with Annexin V + /PI- and Annexin V + /PI + cells was deemed apoptotic cells.

To assess caspase-3 and -7 activities on treatment with ZBC260, the caspase-Glo 3/7 Assay from Promega Corporation (USA) was used^36,37^. Briefly, seeding of indicated cells (5 × 10^3^ cells) was done in 96-well plates and rested overnight. ZBC260 with or without z-VAD-fmk pretreatment was added for 24 h in triplicate, and the assay Caspase-Glo^®^ 3/7 was conducted as per provided indications in three experiments conducted independently.

### Assay for colony formation

A total of 1000 cells in 2 mL complete medium were seeded in six-well plates with flat-bottoms. After 24 h, treatment of cells with ZBC260 for another 24 h. Further culture was performed for 2 weeks at 5% CO_2_ and 37 °C. Staining was performed with crystal violet following a fixation step with methanol.

### Assay for Sarcosphere formation

The assay for the formation of Sarcosphere was performed in accordance with a previously described protocol. Briefly, seeding of 1 × 10^3^ cells were done in 6-well plate of ultralow attachment in DMEM culture medium along with human bFGF and human EGF (20 ng/mL each), and N2 medium (Invitrogen). After every 3 days the culture media was changed. Using inverted phase contrast microscopy in three random fields, after a 14-day culture, sarcospheres were quantitated.

Following treatment with compounds for the formation of primary sarcospheres, treatment was not applied to assay the formation of secondary sarcospheres. Every 3 days, changing of culture media was done. After two full weeks of culturing, quantification of sarcospheres was done by inverted phase contrast microscopy (three fields chosen randomly).

### β-catenin overexpression

A full-length human β-catenin was PCR amplified and cloned into the pcDNA3.1/V5-His vector from Invitrogen. Transfection of U87 and U251 cells was done with Lipofectamine 2000 from Invitrogen as per provided instructions.

### Immunohistochemical (IHC) analysis

The IHC was performed base on the manufacturer instruction using antibodies against Ki-67, Bcl-2, proliferating cell nuclear antigen (PCNA), GLI family zinc finger 1 (GLI1), NOTCH1 intracellular domain (NICD1), and β-catenin. The evaluation and scoring of staining intensity were done by two pathologists independently. The extent of staining was scored as follows: 0: 0% cells stained; 1: 1–25% cells stained; 2: 26–50% cells stained; 3: 51–75% cells stained; 4: >75% cells stained. Intensity of staining intensity was scored as follows: 0, negative; 1, weak; 2, intermediate or 3, strong. The product of the intensity and extent scores determined the final staining score. Staining scores over 6 were considered representative of high levels of expression.

### Real-time PCR

Real-time PCR was conducted as previously described^38,39^. RNA was isolated from controls (vehicle treatment) and experimental (ZBC260) with group TRIzol reagent (Invitrogen) following the provided instructions. The PrimeScript^TM^ RT Reagent Kit (Takara) was used for cDNA synthesis. Real-time PCR amplification was carried using SYBR^®^ Premix (Takara) on a Real-Time PCR System (ABI ViiATM 7Dx). The expression of β-actin was used to normalize expression of relative gene, which was determined through the 2^–ΔΔCt^ method. The primers are listed in Table 1.

**Table 1:** The primers sequence.

	Forward	Reverse
p21	5ʹ-CATGTGGACCTGTCACTGTCTTGTA-3ʹ	5ʹ-GAAGATCAGCCGGCGTTTG-3ʹ
cyclin D1	5ʹ-ATGTTCGTGGCCTCTAAGATGA-3ʹ	5ʹ-CAGGTTCCACTTGAGCTTGTTC-3ʹ
cyclin B1	5ʹ-AATGAAATTCAGGTTGTTGCAGGAG-3ʹ	5ʹ-CATGGCAGTGACACCAACCAG-3ʹ
p27	5ʹ-AAGCGACCTGCAACCGACGATTCTT-3ʹ	5ʹ-GCTCCACAGAACCGGCATTT-3ʹ
β-actin	5ʹ-CATCACCATTGGCAATGAGC-3ʹ	5ʹ-CATACTCCTGCTTGCTGATC-3ʹ
ALDH1	5ʹ-GACAGGCTTTCCAGATTGGCTC-3ʹ	5ʹ-AAGACTTTCCCACCATTGAGTGC-3’
c-Myc	5ʹ-TGACCTAACTCGAGGAGGAGCTG-3ʹ	5ʹ-AAGTTTGAGGCAGTTAAAATTATGG-3ʹ
KLF4	5ʹ-GAACTGACCAGGCACTACCG-3ʹ	5ʹ-TTCTGGCAGTGTGGGTCATA-3ʹ
Nanog	5ʹ-ACATGCAACCTGAAGACGTGTG-3ʹ	5ʹ-CATGGAAACCAGAACACGTGG-3ʹ
Sox2	5ʹ-GCATTCAAACTGAGGCACCA-3ʹ	5ʹ-AAATGGGAGGGGTGCAAAAG-3ʹ
ABCG2	5ʹ-TTTCCAAGCGTTCATTCAAAAA-3ʹ	5ʹ-TACGACTGTGACAATGATCTGAGC-3ʹ
AXIN2	5ʹ-ACTGCCCACACGATAAGGAG-3ʹ	5ʹ-CTGGCTATGTCTTTGGACCA-3ʹ
MMP7	5ʹ-TAGGCGGAGATGCTCACTTT-3ʹ	5ʹ-TTCTGAATGCCTGCAATGTC-3ʹ
PPARD	5ʹ-ACTGAGTTCGCCAAGAGCATC-3ʹ	5ʹ-ACGCCATACTTGAGAAGGGTAA-3ʹ
HES1	5ʹ-ACACCGGACAAACCAAAGAC-3ʹ	5ʹ-AATGCCGGGAGCTATCTTTC-3ʹ
CCND3	5ʹ-GCTTCTCCTAGGACTCGCTAAC-3ʹ	5ʹ-CATGTGCGGCTTGATCTCCT-3ʹ
GLI1	5ʹ-TCTCAAAGTGGGAGGCACAA-3ʹ	5ʹ-CCCTTAGGAAATGCGATCTG-3ʹ
HHIP	5ʹ-CGAAACATGAGAGGCTGTGT-3ʹ	5ʹ-CGCTGGGCTCTAGATGATG-3ʹ

### Western blotting

Western blotting was performed as previously described^40,41^. Total protein was prepared using RIPA lysate buffer. Then, 50 μg of protein was resolved using 12% sodium dodecyl sulfate polyacrylamide gel electrophoresis. Polyvinylidene fluoride (PVDF) membranes were used for transfer and subjected to blocking with nonfat milk (5%) with shaking for 24 h and incubated with primary antibodies at 4 °C overnight. This was followed by 10 min washing with PBS Tween-20 (PBST) thrice and incubation with 1: 4000 dilution of horseradish peroxidase (HRP)-conjugated goat anti-rabbit antibody (Cell Signaling Technology, USA) for 60 min. This was followed by PBS Tween-20 (PBST) three washes for 10 min each. A 1:1 solution of luminol and peroxide solution (Millipore, Billerica, MA, USA) was used for detection of proteins and subsequent image capture. The primary antibodies are list as follows: E-cadherin (#14472, Cell Signaling Technology), CD44 (#3570, Cell Signaling Technology), SNAI2 (#9585, Cell Signaling Technology), vimentin (#5741, Cell Signaling Technology), N-cadherin (#4061, Cell Signaling Technology), p21 (#2947, Cell Signaling Technology), p27 (#3686, Cell Signaling Technology), cleaved PARP (#9548, Cell Signaling Technology), cleaved caspase 3 (#9661, Cell Signaling Technology), c-Myc (#9402, Cell Signaling Technology), Nanog (#8822, Cell Signaling Technology), Sox2 (ab93689, Abcam), PCNA (ab92552, Abcam), Kruppel-like factor 4 (KLF4) (ab215036, Abcam), BRD2 (ab3718, Abcam), BRD3 (ab264420, Abcam), BRD4 (ab128874, Abcam), ALDH1 (ab129815, Abcam), ATP binding cassette subfamily G member 2 (ABCG2) (ab3380, Abcam), Bcl-2 (ab32124, Abcam), Bcl-X_L_ (ab32370, Abcam), cyclin D1 (sc-8396, Santa Cruz Biotechnology), cyclin B1 (sc-245, Santa Cruz Biotechnology), β-catenin (sc-7963, Santa Cruz Biotechnology), Bax (sc-7480, Santa Cruz Biotechnology), Ki67 (sc-23900, Santa Cruz Biotechnology), GLI1 (sc-515780, Santa Cruz Biotechnology), β-actin (sc-8432, Santa Cruz Biotechnology), and NICD1 (sc-376403, Santa Cruz Biotechnology).

### TOP-flash assay

The TCF/LEF reporter plasmids including TOP-flash and FOP-flash were obtained from Upstate (USA). Briefly, the glioma cells were cotransfected with the reported construct p-TOP-flash for a short duration of time, which contains a LEF/TCF enhancer in the upstream region of luciferase enzyme coding region, or control (pFOP-flash). Glioma cells were used as the transfection internal control, and were cotransfected with a TCF/LEF-independent β-gal vector. Activities of β-gal and luciferase were examined one full day after transfection. The experiments were carried out three times and in triplicates. Determination of gene transcription mediated by TCF was done by pTOP-flash/pFOP-flash luciferase activity ratios, and normalization for each was done relative to the β-gal enzyme activity level. Comparison was made between the mean and the normalized values.

### Xenograft model

The studies received approval from the medical ethical committee of The People’s Hospital of China Medical University (The People’s Hospital of Liaoning Province). The guidelines of the Center of Experiment Animal of The People’s Hospital of China Medical University (The People’s Hospital of Liaoning Province) were followed for studies in mice. Female BALB/c nude mice (4–6-week-old) were subjected to subcutaneous injection with 1 × 10^6^ U251 cells in 200 μL PBS in the vicinity of the scapula. This was followed by random assignment of mice 7 days post injection into a control group that received treatment with vehicles (10% PEG400: 3% Cremophor: 87% PBS, or 2% TPGS:98% PEG200) or a treatment group that received ZBC260 (5 mg/kg) by i.v. injection every other day (*n* = 6). The formula, tumor volume (*V*) = ½ (width^2^ × length) was used to calculate tumor volume with measurements of width and length and was recorded every 3 days along with measurements of body weight. Following sacrifice of the animals with 80 mg/kg pentobarbital euthanised at day 22, resection was performed with care to calculate tumor weights. Tumors were embedded in paraffin after being dissected and fixed in ten percent formalin. Terminal deoxynucleotidyl transferase-mediated dUTP Nick End Labeling (TUNEL, Millipore) and active caspase-3 (#9661, Cell Signaling Technology) immunostaining were carried out on 5-μm tumor sections embedded in paraffin as described previously, using a secondary antibody conjugated with Alexa Fluor 488 for detection.

### Statistical analyses

The mean ± SD was the mode for expressing data from three independent experiments. SPSS software 20.0 was used for two-tailed Student’s *t*-test or log-rank test to analyze variations with statistically significant values at *P* < 0.05.

## Results

### ZBC260 potently suppresses viability of glioma cells

**Fig. 1:**
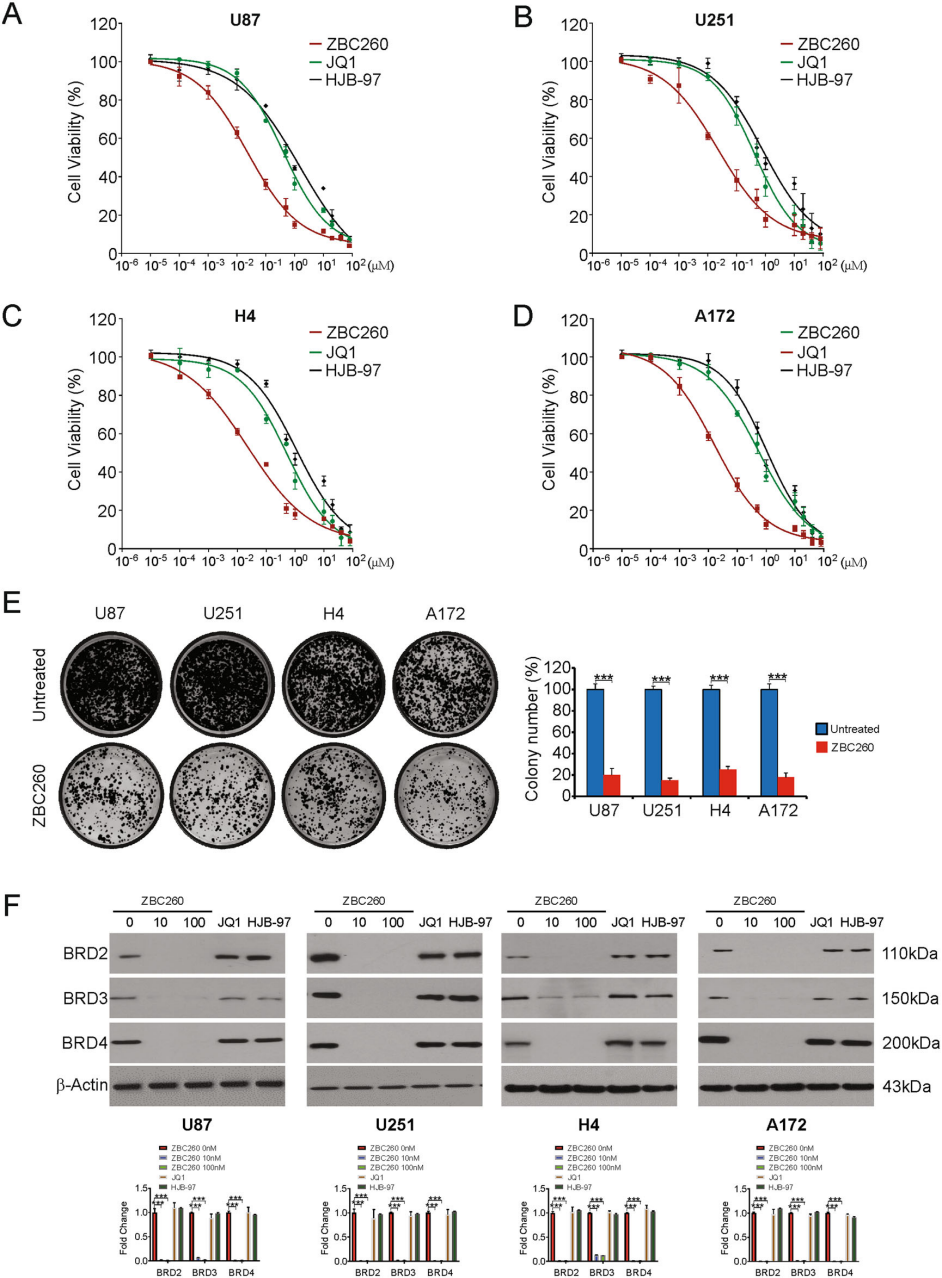
ZBC260 inhibits proliferation and induces cell cycle arrest in glioma cells. **A–D** Indicated glioma cells were treated with increasing dose of ZBC260, JQ1 or HJB-97 for 72 h. The viability of cells was analyzed by the MTT assay. **E** Indicated cells were treated with 100 nM ZBC260 for 24 h. Colony formation assay was done by seeding an equal number of treated cells in 6-well plates, and then staining attached cells with crystal violet 14 days later. **F** Indicated cells were treated ZBC260 at indicated concentration, 10 μM JQ1 or 10 μM HJB-97 for 24 h. Indicated proteins level were analyzed by western blotting and normalized to β-actin. Results were expressed as means ± SD of three independent experiments. ***P < 0.001.

To examine the effect of ZBC260 on glioma cells, ZBC260, JQ1, and HJB-97 were administered for 72 h at increasing concentrations to human glioma cell lines to examine the sensitivity of the cancer cells. The viability of all glioma cell lines decreased by ZBC260, associating directly with the dose administered (Fig. 1A–D). Our results also indicated that glioma cells are more sensitive to ZBC260 than JQ1 and HJB-97 in all tested cell lines (Fig. 1A–D). The formation of colonies was analyzed to observe the effect of ZBC260 on the growth of glioma cells. (Fig. 1E). Conclusively, our findings indicate that ZBC260 is more potent than JQ1 and HJB-97 to suppresses the glioma cells proliferation in vitro.

### ZBC260 is a potent BET degrader in glioma cells

We then examined the activity of ZBC260 in degrading BET proteins in the glioma cell lines. Western blotting results showed that ZBC260 potently degraded BRD2/3/4 cells was then analyzed^28^. ZBC260 promotes degradation of BRD2/3/4 in all four glioma cell lines as displayed by Western Blotting (Fig. 1F). In addition, ZBC260 downregulates BRD2/3/4 in a time-dependent manner in U87 cells (Supplementary Fig. S1A). Similar to the data reported previously^24^, there was no impact of BET inhibitors JQ1 and HJB-97 on protein levels of BRD2/3/4 in glioma cells (Fig. 1F). Next, whether the degradation of BET protein was also initiated by ZBC260 via the UPS-dependent pathway by MG-132 and MLN4924, the pan-inhibitors of Cullin-based E3 ligase. The results of western blotting assays reveal that either MG-132 or MLN4924 pre-treatment remarkably abrogated the BET degrading ability of ZBC260 in U87 cells (Supplementary Fig. S1B). These results are in accordance with the hypothesis that ZBC260 could induce preferential degradation of BET proteins.

### ZBC260 promotes cell cycle arrest and induces apoptosis in glioma cells

**Fig. 2:**
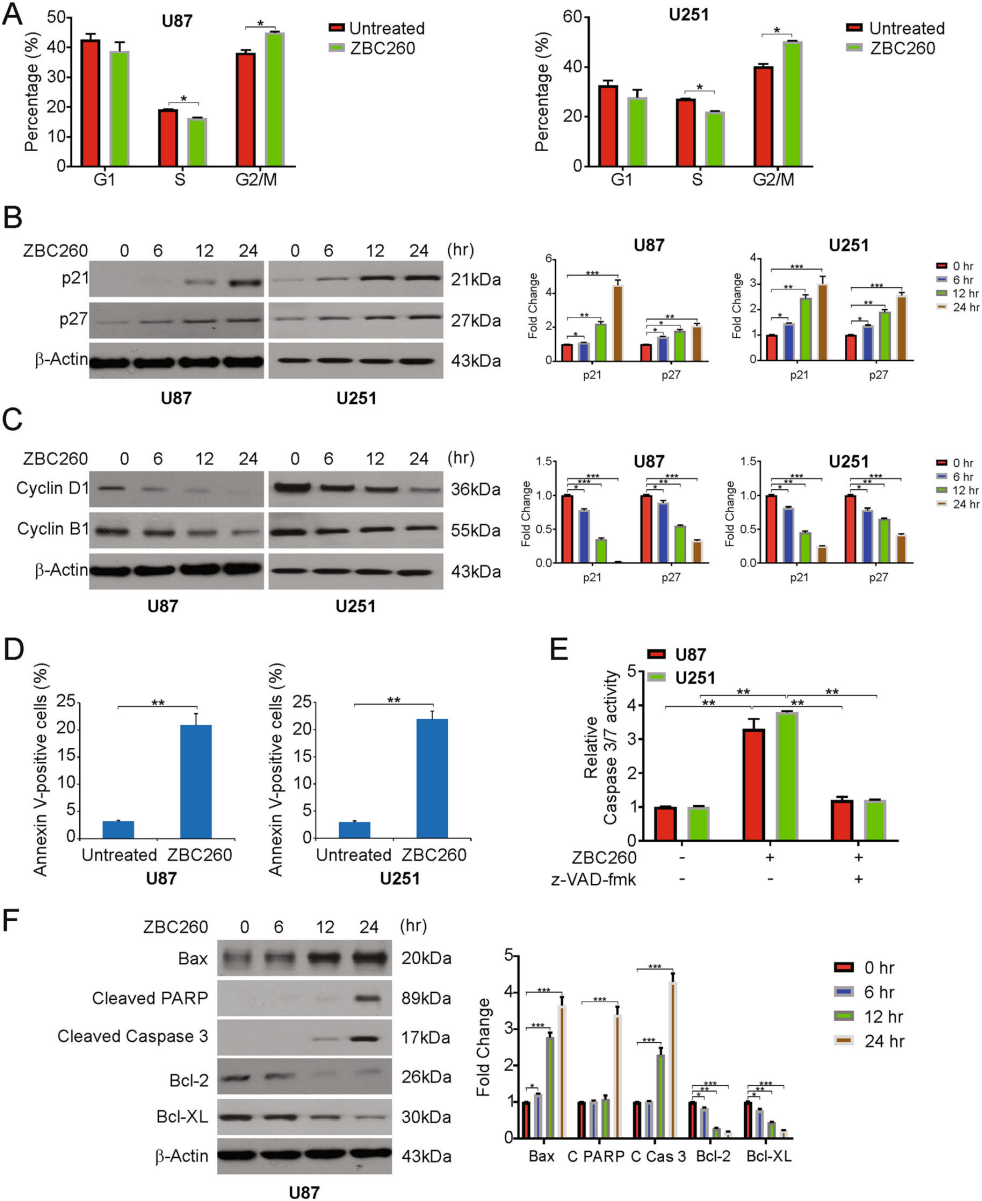
ZBC260 induces cell cycle arrest and apoptosis in glioma cells. **A** Indicated cell lines were treated with 100 nM ZBC260 for 24 h. Cell cycle was analyzed by flow cytometry. **B** Indicated cell lines were treated with 100 nM ZBC260 for 24 h. Indicated protein level was analyzed by western blotting and normalized to β-actin. **C** Indicated cell lines were treated with 100 nM ZBC260 for 24 h. Indicated protein level was analyzed by western blotting and normalized to β-actin. **D** Indicated cell lines were treated with 100 nM ZBC260 for 24 h. Apoptosis was analyzed by flow cytometry. **E** U87 cells were treated with 100 nM ZBC260 for 24 h with or without z-VAD-fmk pretreatment. Caspase 3/7 activity was analyzed by the caspase 3/7 activation kit. **F** U87 cells were treated with 100 nM ZBC260 for 24 h. Indicated protein level was analyzed by western blotting and normalized to β-actin. Results were expressed as means ± SD of three independent experiments. *P < 0.05; **P < 0.01; ***P < 0.001.

Next, the effect of ZBC260 on the cell cycle was studied by exposing the U87 and U251 cells to ZBC260 and subjecting to flow cytometry. The cells were arrested in the G2/M phase (Fig. 2A). Furthermore, we analyzed the levels of p21 and p27, effective inhibitors of cell cycle progression, and found that p21 and p27 mRNA and protein levels increased after treatment with ZBC260 (Fig. 2B and Supplementary Fig. S2A). Cyclin D1 and cyclin B1 were repressed by ZBC260 treatment (Fig. 2C and Supplementary Fig. S2B). Thus, our results demonstrate that ZBC260 promotes cell proliferation inhibition and cell cycle arrest in glioma cells.

In addition, efficient induction of apoptosis of glioma cells by ZBC260 was investigated. The proportion of cells undergoing apoptosis was higher after treatment with ZBC260, as shown by Annexin V/PI staining (Fig. 2D). U87 and U251 cells were treated with ZBC260 with or without pretreatment of a pan-caspase inhibitor, z-VAD-fmk, and analyzed caspase-3/7 activity. Our findings demonstrated that ZBC260 increases the activity of caspase-3/7. However, the caspase-3/7 activity enhancement was attenuated by z-VAD-fmk pretreatment (Fig. 2E). Moreover, our findings indicated that ZBC260 reduced the levels of Bcl-2 and Bcl-X_L_. However, ZBC260 treatment increased the level of Bax, as well as the cleavage of PARP, cleaved caspase-3, and caspase-9 (Fig. 2F and Supplementary Fig. S2C). Taken together, our findings indicate that ZBC260 induces apoptosis in glioma cells.

### ZBC260 inhibited invasion, migration, and EMT in glioma cells

**Fig. 3:**
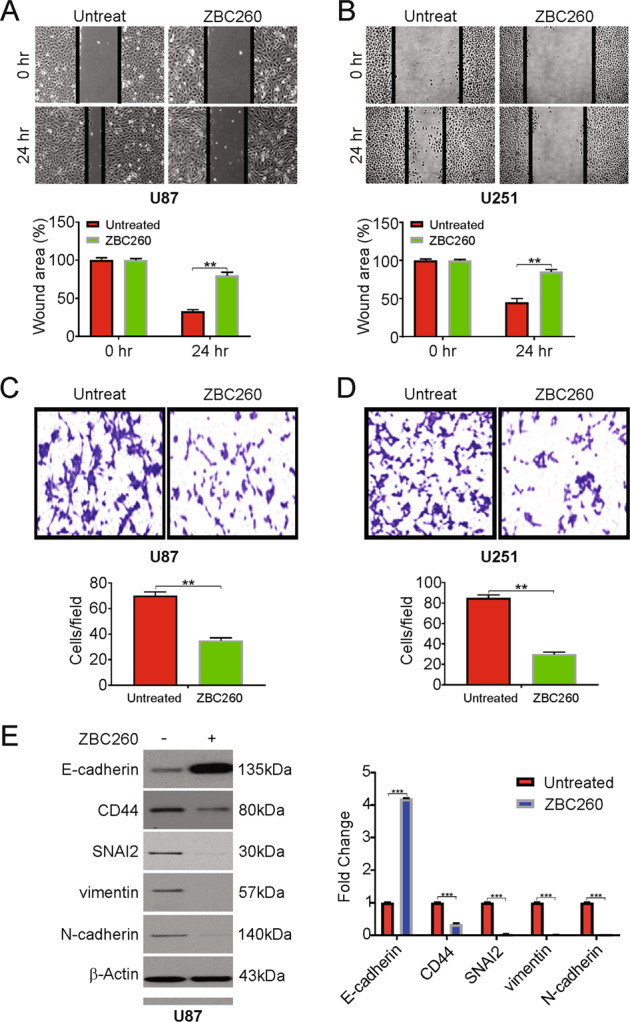
ZBC260 regulates migration, invasion, and EMT in glioma cells. **A** Representative results of wound healing assay showing the effect of ZBC260 (100 nM) treatment on the migration ability in U87 cells. **B** Representative results of wound healing assay showing the effect of ZBC260 (100 nM) treatment on the migration ability in U251 cells. **C** Representative results of Transwell assay showing the effect of ZBC260 treatment invasive ability in U87 cells. **D** Representative results of Transwell assay showing the effect of ZBC260 treatment invasive ability in U251 cells. **E** U87 cells were treated with 100 nM ZBC260 for 24 h. Indicated protein level was analyzed by western blotting and normalized to β-actin. Results were expressed as means ± SD of three independent experiments. **P < 0.01; ***P < 0.001.

Next, we investigated the function of ZBC260 on cell migration, invasion and EMT in glioma cells. The wound-healing assay and the Transwell invasion assays displayed that ZBC260 noticeably contains glioma cell migration and invasion, respectively (Fig. 3A–D). We also evaluated the expression levels of the epithelial marker E- cadherin, N-cadherin, SNAI2, CD44, and vimentin in treated U87 and U251 cells to examine whether EMT is regulated by ZBC260 in glioma cells. Indeed, ZBC260 reduced markedly N-cadherin, SNAI2, CD44, and vimentin expression in both the U87 and U251 cells, while E-cadherin was noticeably upregulated in contrast to the controls (Fig. 3E and Supplementary Fig. S3A, B). In summation, ZBC260 suppresses the invasion, migration, and EMT in glioma cells.

### ZBC260 inhibits the tumor growth of glioma in vivo

**Fig. 4:**
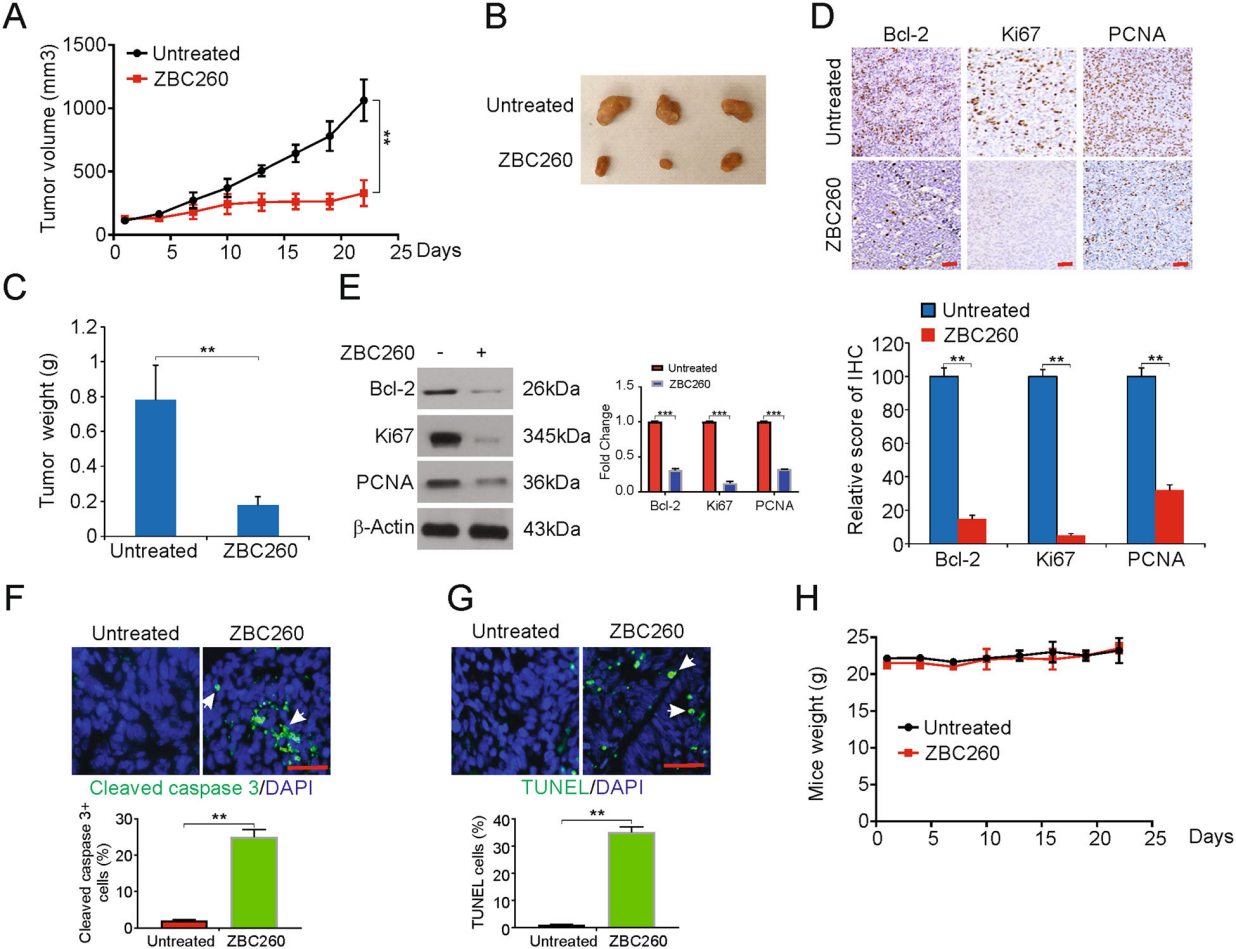
ZBC260 inhibits the growth of xenograft in mouse model. **A** Nude mice were injected s.c. with U251 cells. After one week, mice were treated with ZBC260 or vehicle control. Tumor volume at indicated time points post treatment was calculated and plotted with *P-*values, *n* = 6. **B** Representative tumors at the end of the experiment in **A**. **C** Tumor weight at the end of the experiment in **A**. **D** Paraffin-embedded sections of tumor tissues from mice were analyzed by IHC for Bcl2, Ki67, and PCNA. *Lower*, The score of IHC staining. **E** Western blotting of Bcl2, Ki67, and PCNA in tumor tissues from the experiment in **A**. The western blotting was normalized to β-actin (**F**) Paraffin-embedded sections of tumor tissues from mice were analyzed by cleaved caspase 3 staining. **G** Paraffin-embedded sections of tumor tissues from mice were analyzed by TUNEL staining. **H** Effect of ZBC260 on the body weight of mice over the treatment time. Results were expressed as means ± SD of three independent experiments. **P < 0.01; ***P < 0.001.

The inhibitory effect of ZBC260 was shown clearly in vitro. We then examined the effects of ZBC260 in vivo. U251 cells were subcutaneously injected into nude mice, and the mice were monitored until the tumor volume was nearly 50 mm^3^. Then, the mice were categorized into vehicle and ZBC260 groups. As seen in the tumor growth curves, mice exposed to ZBC260 treatment exhibited decreased tumor size and weight by the end of the study (Fig. 4A–C). These results suggest an in vivo decrease in the growth of glioma in response to ZBC260.

The extent of apoptosis and the presence of inhibitory effects of ZBC260 in glioma in vivo were assayed by immunohistochemistry and western blotting of tumor samples. The levels of Ki-67, Bcl-2, and PCNA decreased in tumors exposed to ZBC260 (Fig. 4D–E). In addition, treatment of ZBC260 induced tumors apoptosis, as shown by cleaved caspase-3 and TUNEL assay staining (Figs. 4F, G). The difference in the average body weight of mice treated with vehicles or ZBC260 was not significant (Fig. 4H). These results, thus, demonstrate that ZBC260 exhibits antitumor properties in human glioma cells, without severe toxicity or side effects.

### ZBC260 suppressed the stem-cell-like features of glioma cells

**Fig. 5:**
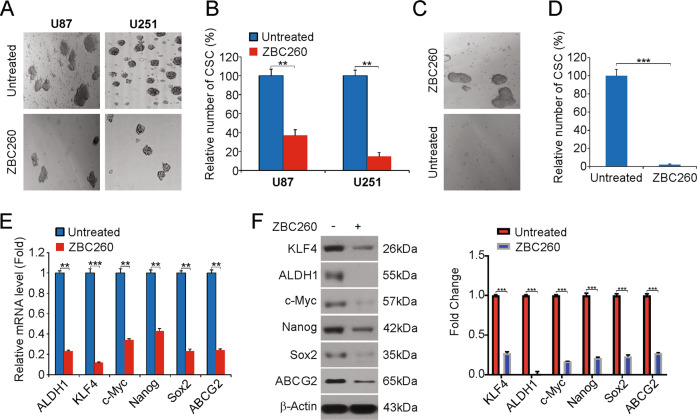
ZBC260 inhibits stem-cell-like properties of glioma cells. **A**, **B** Sarcosphere-formation capacity of indicated cells was analyzed post 100 nM ZBC260 treatment. **C**, **D** Secondary sarcosphere formation capacity of U251 cells was analyzed without further treatment after 100 nM ZBC260 treatment in primary sarcosphere-formation assay. **E** mRNA expression of stem cell markers in U251 cells treated with 100 nM ZBC260 for 24 h was examined by Real-time RT-PCR. **F** U251 cells were treated with 100 nM ZBC260 for 24 h. The protein level of stem cell markers was analyzed by western blotting and normalized to β-actin. Results were expressed as means ± SD of three independent experiments. **P < 0.01; ***P < 0.001.

Success in the clinic is hampered by a major fraction of patients showing decreased sensitivity or increased resistance to drugs used for therapy. The success of therapy can be thwarted by the features of cancer stem cells (CSCs), including self-renewal and maintenance of tumor characteristics. The results shown above highlight the in vitro and in vivo inhibition of glioma by ZBC260, so we next proceeded to examine the possibility of inhibition of stem-cell-like features of these cancer cells. U87 and U251 cells exposed to ZBC260 produced fewer sarcospheres (Figs. 5A, B), as shown by the assay for the formation of sarcospheres. To analyze whether the self-renewal property of glioma stem cells was affected by ZBC260, an assay for the formation of secondary sarcospheres was performed. While the U251 cells exposed to vehicles formed secondary sarcospheres, U251 cells exposed to an initial ZBC260 treatment followed by no subsequent treatment that did not form secondary sarcospheres (Fig. 5C, D).

The levels of stem cell markers following treatment with ZBC260 were determined in U87 and U251 cells. Major markers, such as Aldehyde dehydrogenase 1 (ALDH1), KLF4, SOX2, NANOG, c-Myc, and ABCG2, were significantly reduced with exposure to ZBC260 (Fig. 5E, F and Supplementary Fig. S4A, B). Overall, the stem-like features of cells, such as self-renewal, were reduced by ZBC260.

### Targeting of the Wnt/β-catenin pathway to inhibit the stemness of glioma stem cell-like features by ZBC260

**Fig. 6:**
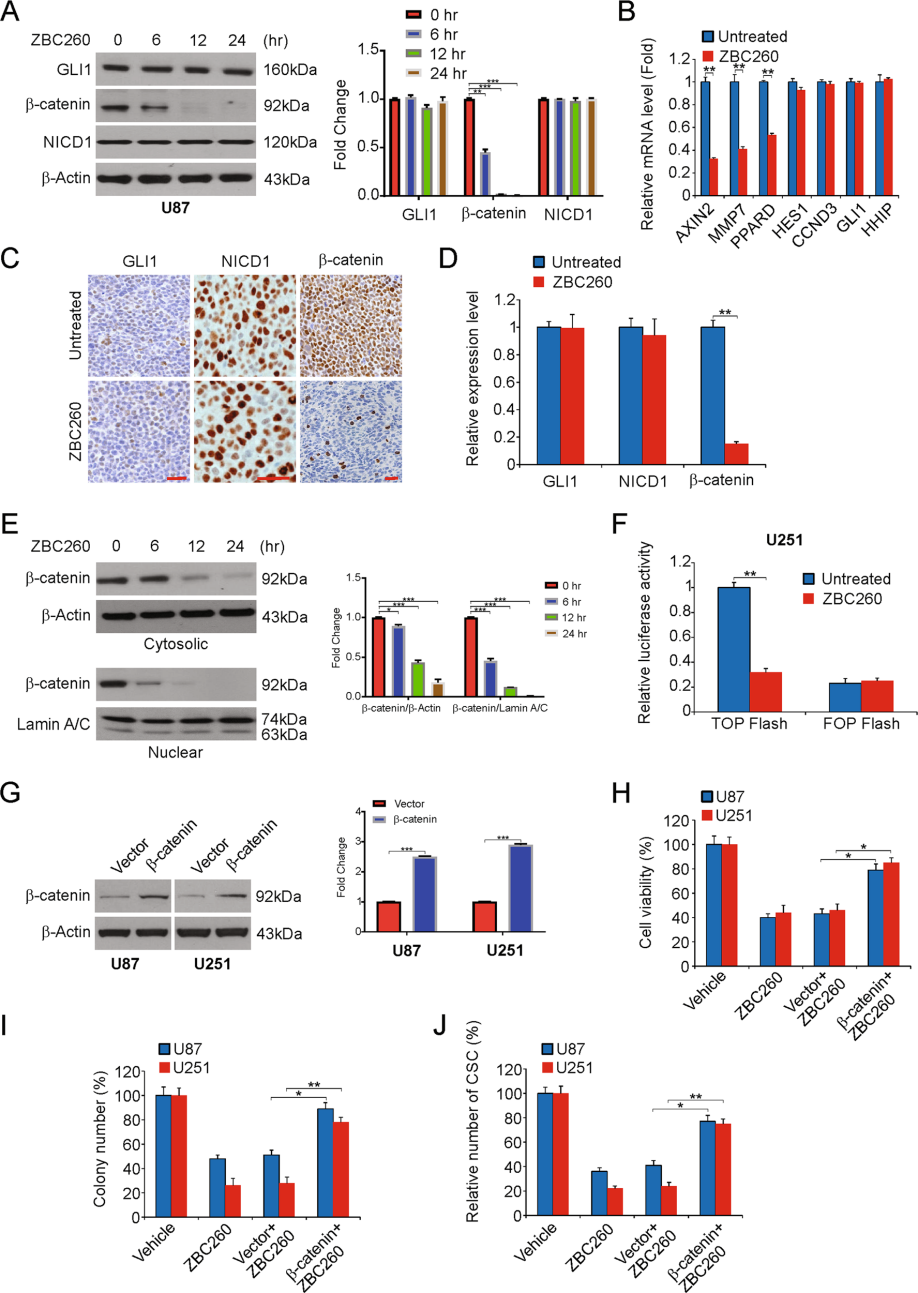
ZBC260 regulates glioma-cell-like properties via Wnt/β-catenin pathway. **A** U87 cells were treated with 100 nM ZBC260 at indicated time point. The expression of β-catenin, NICD1, and GLI1 involved in CSC pathways was analyzed by western blotting and normalized to β-actin. **B** U251 cells were treated with 100 nM ZBC260 for 24 h. mRNA level of target genes was analyzed by Real-time RT-PCR. **C** Paraffin-embedded sections of tumor tissues from mice were analyzed by IHC for β-catenin, NICD1 and GLI1. **D** The score of IHC staining. **E** U251 cells were treated with 100 nM ZBC260 at indicated time point. The level of cytosolic and nuclear β-catenin was analyzed by western blotting and normalized to β-actin or Lamin A/C as indicated. **F** The activity of TCF/β-catenin reporter (TOP/FOP Flash) in 100 nM ZBC260-treated U251 cells. **G** The efficiency of β-catenin overexpression was analyzed by western blotting and normalized to β-actin. **H** Indicated cell lines transfected with β-catenin were treated with 100 nM ZBC260 for 72 h. The viability of cells was analyzed by the MTT assay. **I** Indicated cell lines transfected with β-catenin were treated with 100 nM ZBC260 for 24 h. Colony formation assay was done by seeding anequal number of treated cells in 6-well plates, and then staining attached cells with crystal violet 14 days later. **J** Indicated cell lines transfected with β-catenin were treated with 100 nM ZBC260 for 24 h. Sarcosphere-formation capacity was analyzed. Results were expressed as means ± SD of three independent experiments. *P < 0.05; **P < 0.01; ***P < 0.001.

To elucidate the mechanism by which ZBC260 inhibits the stem cell features of cancer cells, the major CSC signaling pathways Notch, Wnt, and Hedgehog were evaluated primarily in our study. We analyzed the levels of GLI1, NICD1, and β-catenin involved in CSC pathways. Of these, the level of β-catenin decreased with exposure to ZBC260 in accordance with the dose administered. However, NICD1 and GLI1 showed no significant changes (Fig. 6A and Supplementary Fig. S5A). Then, a few target genes downstream in these pathways were examined. In line with the above results, the levels of axis inhibition protein 2 (AXIN2), peroxisome proliferator-activated receptor delta (PPARD), matrix metallopeptidase 7 (MMP7), all associated with the Wnt/β-catenin pathway, were reduced significantly, while downstream targets of the other two pathways, HHIP/GLI1 of Hedgehog and CCND3/HES1 of Notch, were not significantly affected (Fig. 6B and Supplementary Fig. S5B).

Immunohistochemical analysis for NICD1, β-catenin, and GLI1 was performed to confirm the in vivo results in the ZBC260-exposed tumor cells of mice. In addition, our findings suggest that such tumors had a significant decline in β-catenin levels, but not GLI1 or NICD1 levels, when compared to those in the control group (Fig. 6C, D).

Verification of decreased Wnt/β-catenin pathway signaling in glioma by ZBC260 was performed by detecting the amounts of β-catenin (cytosolic and nuclear) by western blotting. These experiments revealed a decline in the levels of nuclear and cytosolic β-catenin with even more reduction in the levels of the former (Fig. 6E and Supplementary Fig. S5C). The TOP/FOP Flash assay, which is a well-known dual-luciferase reporter assay for TCF/β-catenin was used in analyzing the effect of ZBC260 on the activity of β-catenin signaling. TCF-responsive sites are contained in the TOP Flash reporter. On the other hand, the mutant TCF binding sites are contained in the FOP Flash reporter, which serves as a negative control. Results therefore clearly indicate that following the treatment of U251 cells using ZBC260 the TOP Flash luciferase activity noticeably reduced (Fig. 6F and Supplementary Fig. S5D). Therefore, the above findings indicate that ZBC260 regulates glioma stem cell-like properties via the pathway for Wnt/β-catenin signaling.

We then studied the role of Wnt/β-catenin signaling in the growth and affected stem cell-like features of glioma by ZBC260. With β-catenin overexpression in the cells, a partial rescue was observed for the same factors (Fig. 6G–J). Thus, our findings demonstrated that the Wnt/β-catenin pathway repression participates in the inhibition of stem cell features in response to ZBC260.

## Discussion

Chemotherapy, radiotherapy, and surgical resection are being used to treat gliomas, the most common primary brain tumor, and this has seen outcomes of severe disability and morbidity^42^. While the overall mortality rate of high-grade gliomas remains pressimistic, many studies are being conducted in the realm of molecular studies with the aim to develop new strategies therapeutically. Thus, the identification of the mechanisms that underlie the development of glioma and the identification of new therapeutic targets should be given high priority. It is imperative that a search be performed for new agents to target glioma with increased efficiency yet diminished or absent side effects. The current study shows that the growth of tumors and the stem cell-like properties of glioma were reduced by ZBC260 via inhibition of the β-catenin pathway both in vitro and in mouse models.

A number of studies have found that BRD4 inhibitors lead to the accumulation of BRD4 protein in cancer cells^43,44^. This increase of BRD4 levels, together with the reversible nature of inhibitor binding, could prevent efficient BRD4 inhibition^44^. One strategy to achieve more effective BRD4 inhibition is to design BRD4 degraders, which have received noticeable attention in the past few years, as they may achieve the desired pharmacological effect at lower drug concentrations. PROTAC molecules are a unique compound family and recruit a ubiquitin ligase after binding their target proteins, to promote the targeted degradation of the protein^45^. In the battle against colorectal, prostrate, and breast cancer, PROTACs targeting the BET molecule has reported great anticancer activity^23^. The anti-glioma activity of BET-PROTAC has been analyzed in-depth to find increasingly effective treatment techniques for glioma. ZBC260, along with its corresponding BET inhibitor HJB-97, and JQ1, a prototype BET inhibitor, demonstrates far superior anticancer activity against breast cancer^28,46^. As far as we know, this is the first research that has disclosed the therapeutic benefit of BET-PROTAC in glioma. The findings of this research may be used as supportive evidence for the subsequent purpose of testing these novel agents in the management of glioma.

The success of therapy can be thwarted by features of cancer stem cells (CSCs) such as self-renewal and maintenance of tumor characteristics^47^. The presence of CSCs in glioma has been pointed out, which can help design and test new therapies for glioma^48,49^. Despite surgery and chemotherapy, 70% of patients are resistant to chemotherapy This resistance can be traced back to the CSCs, and the relevant research required this aspect to be kept in mind when designing new strategies^50^. The development of promising treatments for glioma especially in a clinical scenario can involve drugs or molecules that augment CSC sensitivity to chemotherapy or alternatively directly kill these CSCs. A recent study showed that BET inhibitor inhibits CSC properties of CRC cells, and BET inhibitors can reduce the level of CSCs and their stem cell-like features, which suggest that the use of this molecule for targeting CSCs in glioma^17^. The clinical outcome of patients might potentially improve by ZBC260 treatment.

In the past 20 years, there has been great interest in research on pathways responsible for the stem cell-like characteristics of CSCs^47^. This supports the design of treatments that target the major signaling networks in CSCs involving Notch, Hedgehog, and Wnt^47^. The inhibition of glioma CSCs were affected by ZBC260 via repression of Wnt/β-catenin over the other two pathways mentioned. Although in our report and other reports, the exact importance of Wnt/β-catenin in glioma is still inconclusive. Other studies have shown this pathway as a therapeutic intervention for glioma. Our results indicate that ZBC260 suppresses CRCs via Wnt/β-catenin signaling pathway activation.

In conclusion, we found that ZBC260 inhibits tumor growth and stem cell characteristics through the Wnt/β-catenin signaling pathway in glioma cells and xenograft model.

## Supplementary information

Supplementary Figure Legends

Figure S1

Figure S2

Figure S3

Figure S4

Figure S5

## Data Availability

All data generated or analyzed during this study are included in this published article.
