# Supplementary material for: BET degrader inhibits tumor progression and stem-like cell growth via Wnt/β-catenin signaling repression in glioma cells
Source: Cell Death Dis. 2020 Oct 22;11(10):900. doi: 10.1038/s41419-020-03117-1 (PMC7582157; doi:10.1038/s41419-020-03117-1)
Supplement: Supplementary file 1 — Supplementary Figure Legends [file 41419_2020_3117_MOESM1_ESM.docx]

**BET degrader inhibits tumor progression and stem-like cell growth via Wnt/β-catenin signaling repression in glioma cells**

Tao Tian^1^, Tongqi Guo^2^, Wei Zhen^2^, Jianjun Zou^2^, Fuyong Li^2*^

1. Department of Oncology, Shandong Zaozhuang Municipal Hospital, Zaozhuang City, Shandong Province, China.
2. Department of Neurosurgery, The People's Hospital of China Medical University (The People’s Hospital of Liaoning Province), Shenyang, Liaoning Province, China.

***Corresponding author:**

Fuyong Li,

Department of Neurosurgery, The People’s Hospital of China Medical University (The People’s hospital of Liaoning Province), No.33, Wenyi Road, Shenhe District, Shenyang, 110016, Liaoning Province, PR China.

Email: Lifuyong126@126.com

**Running title:** ZBC260 regulates glioma stem-like cells

**Figure S1. (A)** U87 cells were treated with 100 nM ZBC260 at indicated time points. Indicated proteins level were analyzed by Western blotting and normalized to β-actin. **(B)** U87 cells were treated with 10nM ZBC260 with or without 5μM MG132 or 1μM MLN4924 pretreatment. Indicated proteins level were analyzed by Western blotting and normalized to β-actin. Results were expressed as means ± SD of 3 independent experiments. *, *P* < 0.05; ***, *P* < 0.001.

**Figure S2.** **(A)** Indicated cell lines were treated with 100 nM ZBC260 for 24 hours. The mRNA level of P21 and P27 was analyzed by Real-time RT-PCR. **(B)** Indicated cell lines were treated with 100 nM ZBC260 for 24 hours. The mRNA level of P21 and P27 was analyzed by Real-time RT-PCR. **(C)** U251 cells were treated with 100 nM ZBC260 for 24 hours. Indicated protein level was analyzed by Western blotting and normalized to β-actin. Results were expressed as means ± SD of 3 independent experiments. *, *P* < 0.05; **, *P* < 0.01; ***, *P* < 0.001.

**Figure S3. (A) and (B)** U251 cells were treated with 100 nM ZBC260 for 24 hours. Indicated protein level was analyzed by Western blotting and normalized to β-actin. Results were expressed as means ± SD of 3 independent experiments. ***, *P* < 0.001.

**Figure S4. (A)** mRNA expression of stem cell markers in U87 cells treated with 100 nM ZBC260 for 24 hours was examined by Real-time RT-PCR. **(B)** U87 cells were treated with 100 nM ZBC260 for 24 hours. The protein level of stem cell markers was analyzed by Western blotting and normalized to β-actin. Results were expressed as means ± SD of 3 independent experiments. ***, *P* < 0.001.

**Figure S5. (A)** U251 cells were treated with 100 nM ZBC260 at indicated time point. The expression of β-catenin, NICD1, and GLI1 involved in CSC pathways was analyzed by Western blotting and normalized to β-actin. **(B)** U251 cells were treated with 100 nM ZBC260 for 24 hours. mRNA level of target genes was analyzed by Real-time RT-PCR. **(C)** U87 cells were treated with 100 nM ZBC260 at indicated time point. The level of cytosolic and nuclear β-catenin was analyzed by Western blotting and normalized to β-actin or Lamin A/C as indicated. **(D)** The activity of TCF/β-catenin reporter (TOP/FOP Flash) in 100 nM ZBC260-treated U87 cells. Results were expressed as means ± SD of 3 independent experiments. **, *P* < 0.01; ***, *P* < 0.001.
