# Supplementary figures and images for: BET degrader inhibits tumor progression and stem-like cell growth via Wnt/β-catenin signaling repression in glioma cells
Source: Cell Death Dis. 2020 Oct 22;11(10):900. doi: 10.1038/s41419-020-03117-1 (PMC7582157; doi:10.1038/s41419-020-03117-1)

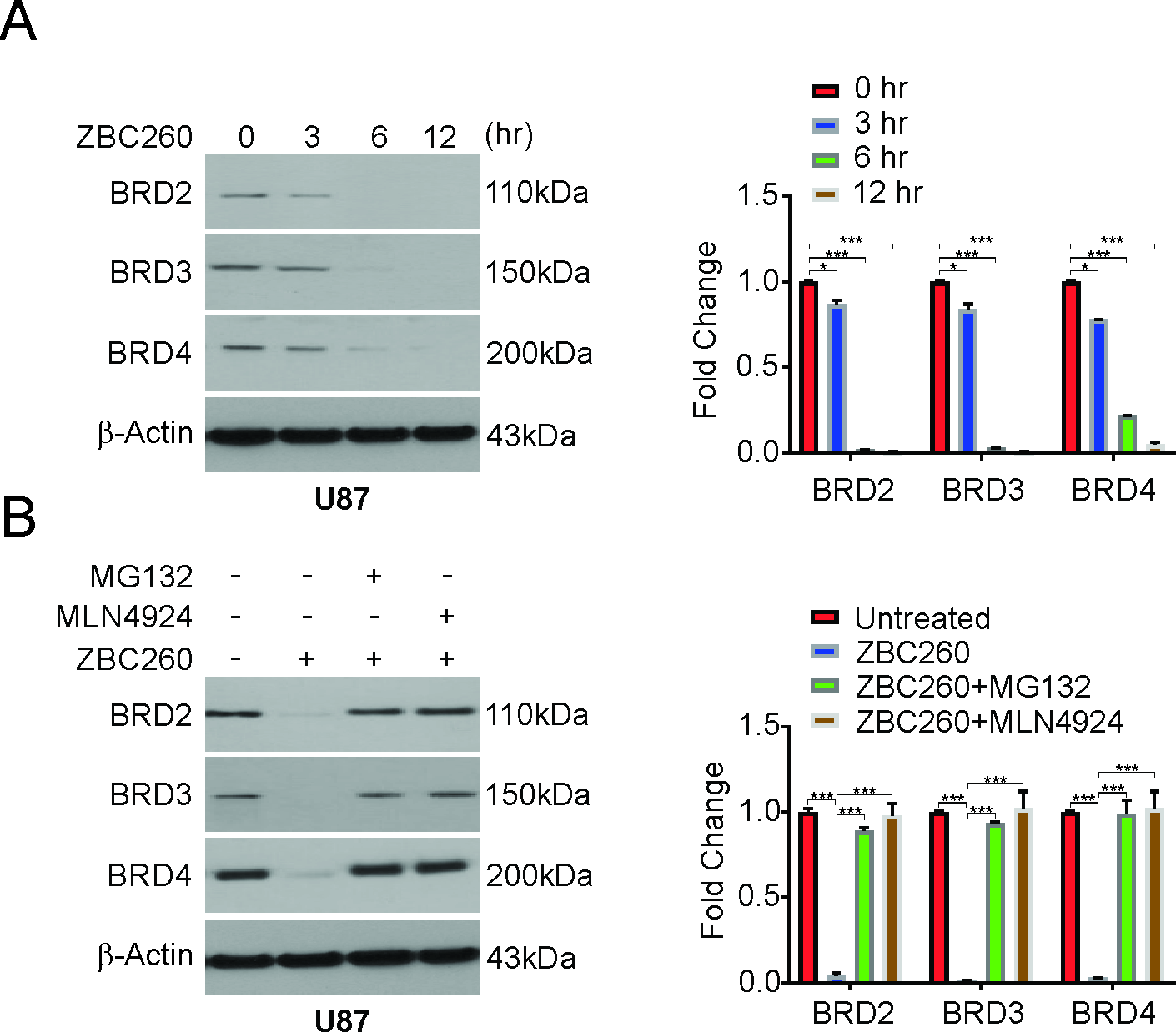

Supplement: Supplementary file 2 — Figure S1 [file 41419_2020_3117_MOESM2_ESM.tif]

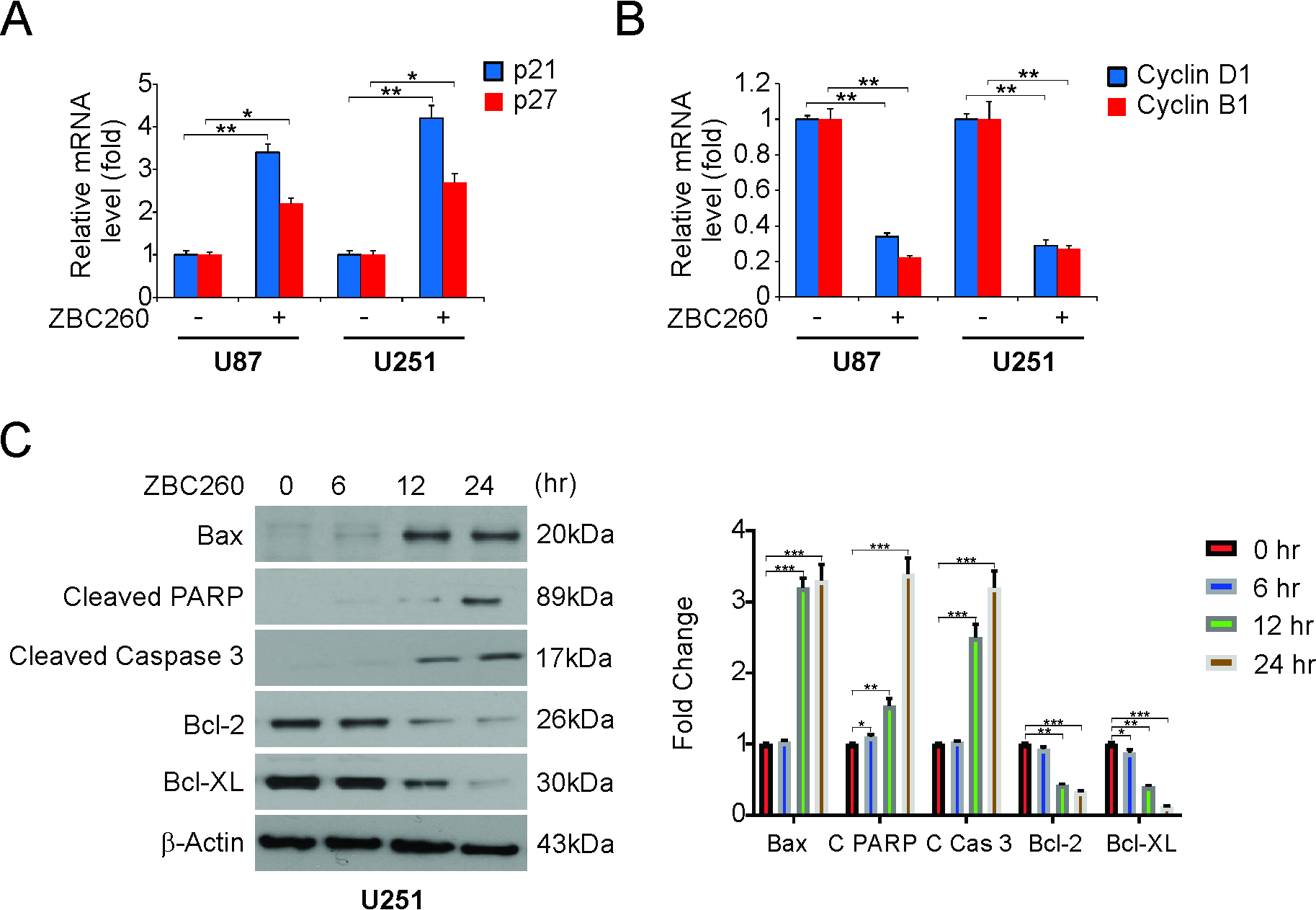

Supplement: Supplementary file 3 — Figure S2 [file 41419_2020_3117_MOESM3_ESM.tif]

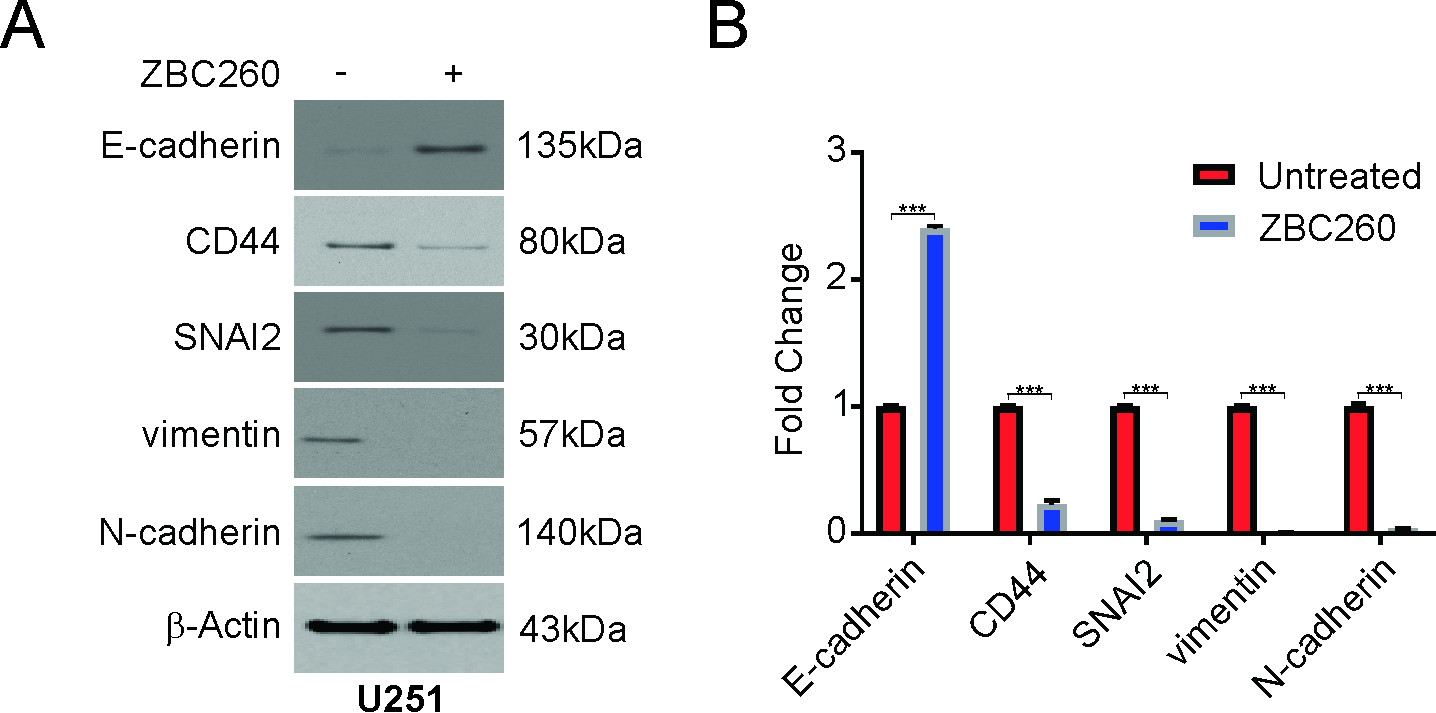

Supplement: Supplementary file 4 — Figure S3 [file 41419_2020_3117_MOESM4_ESM.tif]

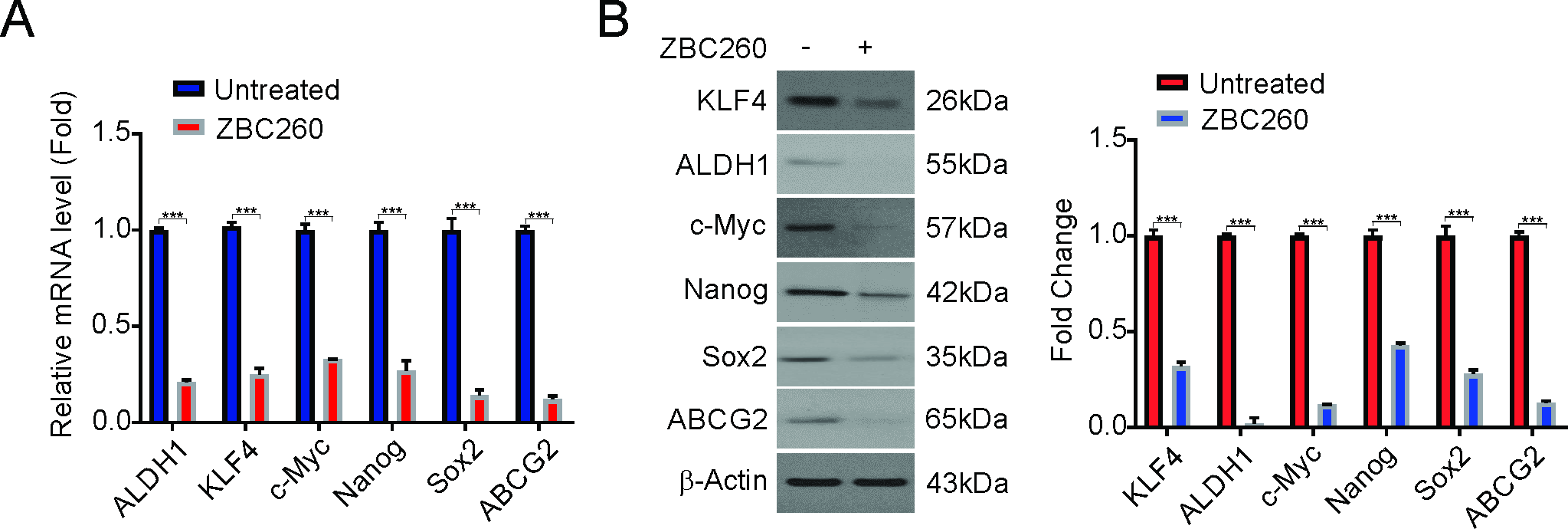

Supplement: Supplementary file 5 — Figure S4 [file 41419_2020_3117_MOESM5_ESM.tif]

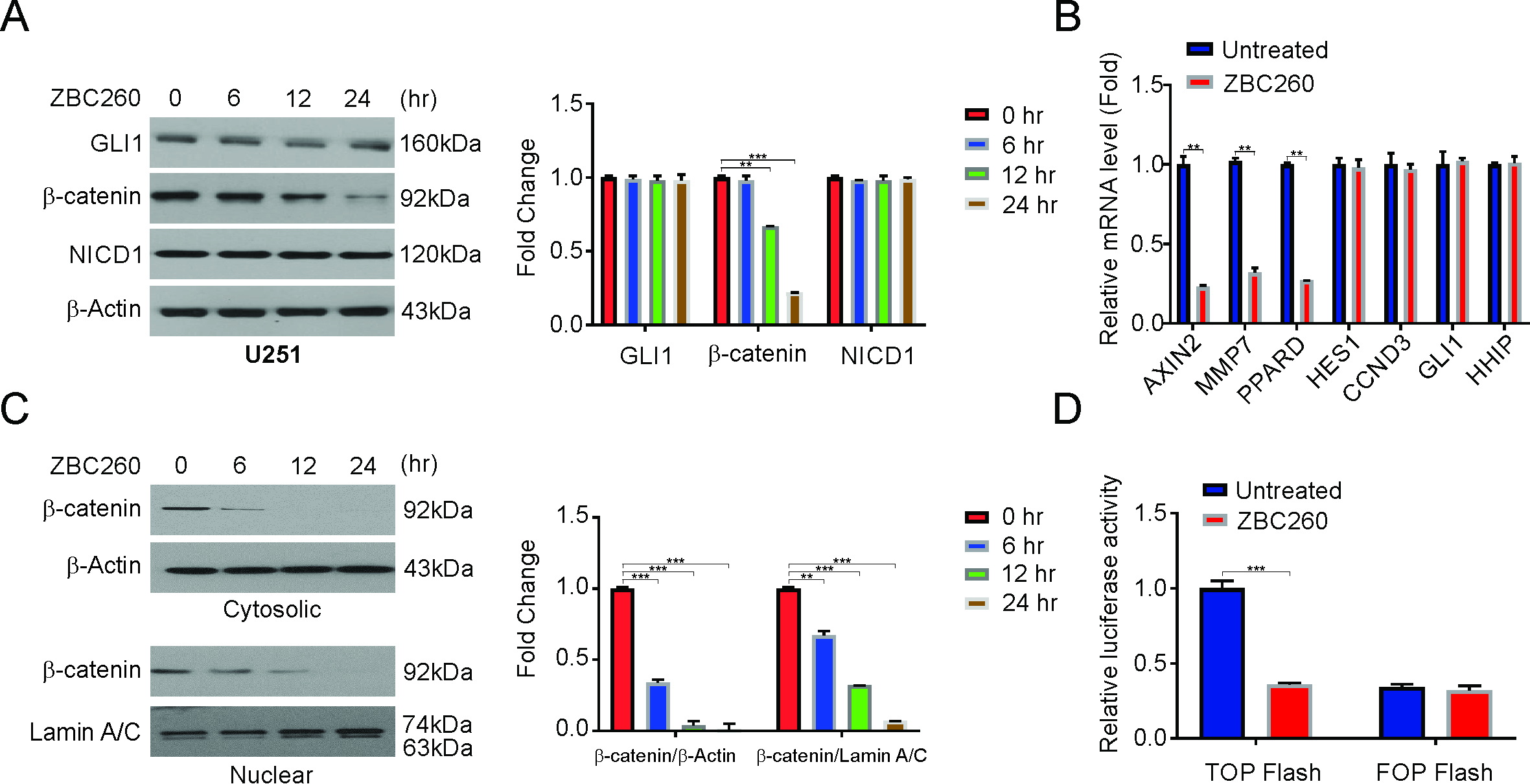

Supplement: Supplementary file 6 — Figure S5 [file 41419_2020_3117_MOESM6_ESM.tif]
